# Light and Scanning Electron Microscopy of Red Blood Cells From Humans and Animal Species Providing Insights into Molecular Cell Biology

**DOI:** 10.3389/fphys.2022.838071

**Published:** 2022-07-01

**Authors:** Gheorghe Benga, Guy Cox

**Affiliations:** ^1^ Romanian Academy, Cluj-Napoca, Romania; ^2^ School of Life and Environmental Sciences, Faculty of Science, University of Sydney, Darlington, NSW, Australia; ^3^ Australian Centre for Microscopy & Microanalysis, University of Sydney, Darlington, NSW, Australia

**Keywords:** erythrocyte, microscopy, marsupial, monotreme, placental mammal, NMR

## Abstract

We reviewed the many discoveries in cell biology, made since the 17^th^ century, which have been based on red blood cells (RBCs). The advances in molecular and structural biology in the past 40 years have enabled the discovery with these cells, most notably, of the first water channel protein (WCP) called today aquaporin1 (AQP1). The main aim of our work reviewed was to examine by light and electron microscopy a very wide range of RBCs from reptiles, birds, monotremes, marsupials and placentals, in order to estimate from these images the RBC cell volume and surface area. The diffusional water permeability of the RBC membrane from these species has further been measured with a nuclear magnetic resonance (NMR) spectroscopy technique. The significance of the observed permeability of RBCs to water and possible influences on the whole body are discussed.

## Red Blood Cells as Objects of Studies in Cell and Molecular Biology Over Centuries

Naked-eye inspection of blood at phlebotomy as part of medical diagnosis was practiced at least 2,000 years ago. However, only after the explosion of interest in microscopy in the 17^th^ century did the examination of the constituents of blood become possible and RBCs were seen. The Dutch microscopist, Antoni van Leeuwenhoek (1632–1723), is credited by many (e.g., De Robertis, 1970) with this discovery. In a critical analysis of the discovery of blood cells, Hajdu, (2003) concluded that the Dutch naturalist Jan Swammerdam (1637-1680) was the first person to observe RBCs under the microscope. However, Antoni van Leeuwenhoek described the size and shape of „red corpuscles” and rendered the first illustration of them in a letter in 1665 to Swammerdam (Letter 42 of Arcana Natura, 1695). The dispute over priority for the initial discovery emphasizes the difficulty in establishing such claims and yet the correct position today is to consider who and when a fact is reported in the formal refereed scientific literature.

Over the centuries, various (and sometimes unexpected) discoveries about living systems have been made in experiments and observations on RBCs. Specifically, it was noted that the sizes of RBCs in various species display much smaller variations compared to the large or even huge differences in body size (mass), considering for example small animals like the mouse or bilby, versus much larger ones like the horse or elephant. Extrapolated to various organs in the body, it became obvious that the total mass of the organ is due to the number and not the volume of each cell. This led to the so called “Law of constant volume”, that was formulated in the 19^th^ century (De Robertis, 1970). The first isolation protocols of nucleic acids were developed in 1869 in Tübingen by the German scientist Friedrich Miescher. He and his superviser, Professor Felix Hoppe-Seyler are now recognized as the discoverers of DNA. They found it in the biological material called „nuclein”. Their work involved RBCs among other cells. In 1871 the first publications of Miescher and Hoppe-Seyler describing the „nuclein” appeared (Miescher, 1871). In addition, another student of Hoppe-Seyler, P. Plósz, (1871), reported the presence of „nuclein” in the hemolyzed nucleated erythrocytes from birds and snakes. The story of the work performed in Tübingen at this time is reviewed by His, (1897). Dahm, (2005) published a more complete history of the discovery of DNA, while Hajdu, (2003) mentioned the discovery in the 19^th^ century of the medical implications of RBCs and the foundation of a new medical specialty, hematology.

The RBC membrane was the main one to reveal the essential features of the structure and function of virtually all cell membranes. Specifically, (1) the „Lipid bilayer model” proposed by the Dutch scientists Gorter and Grendel, (1925); the model proposed in 1935 by the Americans Danielli and Davson, revised in 1943, to include proteins on both surfaces of the lipid bilayer, and also the idea of protein „pores” through the lipid bilayer to allow solute exchange (Davson and Danielli, 1943); the „Fluid mosaic model” proposed in 1972 by Americans Singer and Nicolson: it includes so called „intrinsic membrane proteins” (embedded in the lipid bilayer) and the proteins attached on both sides of the membrane (Singer and Nicolson, 1972); (2) the analysis of membrane proteins by sodium dodecyl sulphate polyacrylamide gel electrophoresis (SDS-PAGE), the analysis of lipids by chromatography, and the study of protein-lipid interactions; (3) the visualization of glycoproteins and glycolipids in the glycocalix; (4) the interactions between the intrinsic membrane proteins and the proteins located inside the cell, in the cytoskeleton; (5) the identification of proteins with roles as antigens (beginning with the blood group antigens). Such aspects are presented in many publications (e.g., De Robertis, 1970; Kummerow et al., 1983; Benga et al., 1984; Benga and Holmes, 1984; Benga et al., 1985a; Benga et al., 1985b; Benga et al., 1985c; Benga et al., 1987a; Benga et al., 1987b; Benga and Tager, 1988; Benga et al., 1989; Benga et al., 1990; Benga et al., 1991; Sperelakis, 2001; Alberts et al., 2008).

## The Transport Processes Across the RBC Membrane and the Discovery of the First Water Channel Protein, Later Called aquaporin1 (AQP1)

The permeability of the RBC membrane to water, ions, micromolecules has been investigated for decades, as reviewed in several books (e.g., House, 1974; Benga et al., 1985a; Benga et al., 1985b; Benga et al., 1985c; Stein, 1986; Benga, 1989a; Benga, 1989b). The start point for the discovery in the RBC membrane of the first water channel protein (WCP), later called aquaporin1 (AQP1), was the comparative NMR measurements of water permeability of the RBC from children with epilepsy and control children performed in 1976 in Cluj-Napoca, Romania, by Gheorghe Benga, Vasile V. Morariu, Ileana Benga and Cornelia Morariu. The results of the study were published in Nature (Benga and Morariu, 1977). The complete story of the discovery was also recently presented (Benga, 2021).

The water permeability of RBCs in children with epilepsy compared with control children was measured by the NMR method of Conlon and Outhred. (1972). The method involves addition of a paramagnetic solution (MnCl_2_) to the plasma and measurement of the spin-spin relaxation time (T_2_) of the RBC water proton. The spin-spin relaxation time of water inside the isolated RBCs is about 140 ms and is much longer than the time required for water to exchange across the membrane (the water exchange time, T_ae_), which is about 10 ms. The value of T_ae_ is inversely related to the water permeability (P_d_) of RBCs. If the relaxation time in plasma is made much shorter than the exchange time (by adding the paramagnetic ion Mn^2+^) the observed relaxation time of the RBC (T_2b_) is dominated by the exchange process through the membrane. The spin-spin relaxation time is evaluated from a logarithmic plot of the nuclear spin-echo as a function of the time interval 2 τ where τ is the time interval between the radiofrequency pulses. When the system is characterized by a single relaxation time, the plot is a straight line and the relaxation time is the reciprocal of the slope. For a system characterized by two relaxation times (as for the blood doped with Mn^2+^) the plot consists of two lines and the relaxation times are calculated from the slopes of these lines; see Figure 1 in ref. Morariu and Benga. (1977), which can be accessed on Google Chrome following two steps:

**FIGURE 1:**
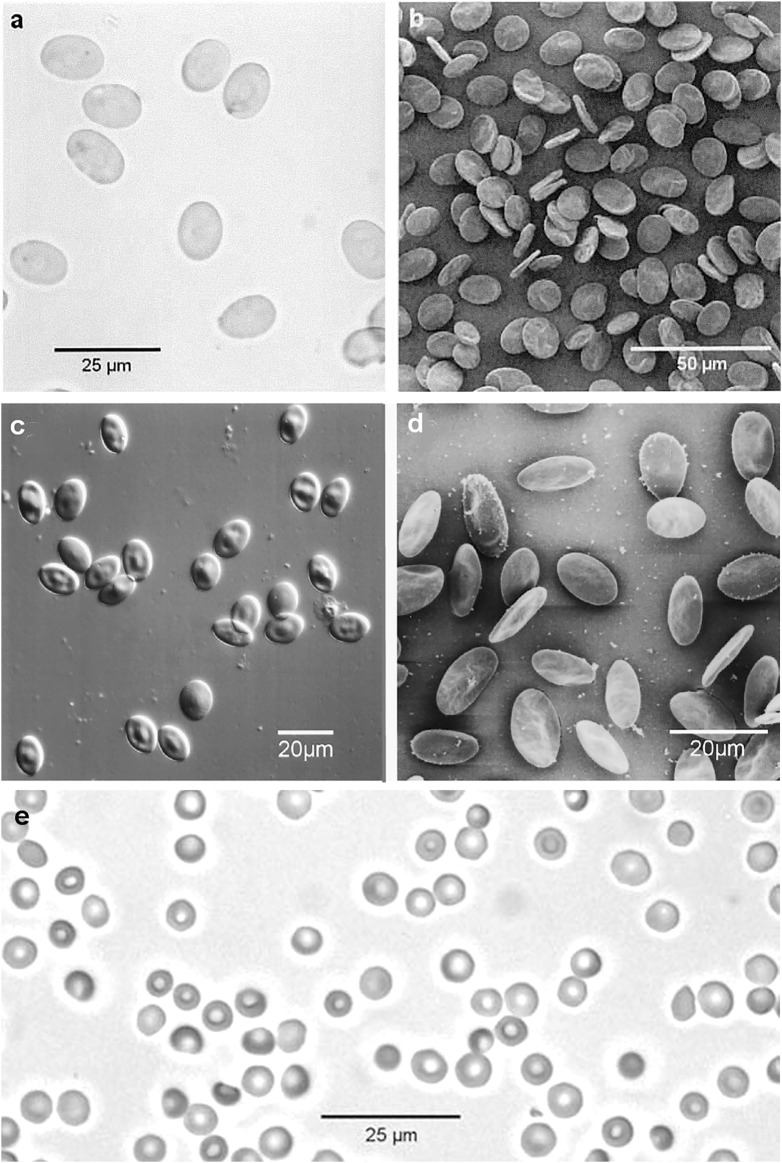
Reptiles, birds and monotremes: **(A)** Green Sea Turtle (*Chelonia mydas*) RBCs, bright field optical micrograph (Nikon Eclipse E800 Plan Apo ×40 0.95NA objective); **(B)** Green Sea Turtle RBCs, secondary electron SEM image (Jeol JSM-6300f); **(C)** Little Penguin (*Eudyptula minor*) RBCs, DIC optical micrograph (Nikon Eclipse E800 Plan Apo ×40 0.95NA objective); **(D)** Little Penguin RBCs, secondary electron SEM image (Jeol JSM-6300f); **(E)** Platypus (*Ornithorhynchus anatinus*) RBCs, bright field optical micrograph. (Nikon Eclipse E800 Plan Apo × 40 0.95NA objective). The original images were published by (Benga*,* 1994; Benga, 2003; Benga et al., 2015).

https://scholar.google.ro/scholar?q=Morariu+Benga+Biochimica+Biophysica+Acta+1977&hl=ro&as_sdt=0&as_vis=1&oi=scholart; then click on [PDF]Academia.edu.

The paper published by Benga and Morariu, (1977) can also be accessed on Google Chrome following two steps: https://af.booksc.eu/book/10454413/a8b701; followed by click on [PDF]. The values of T_ae_ were measured in 24 children with epilepsy (aged 1–12 years) and 24 controls (children aged 2–16 years). In all children with epilepsy the exchange time of water through the RBC membrane (T_ae_) was longer than in control subjects. In other words decreased values of the water permeability were found in case of RBCs from children with epilepsy. There were no significant differences in T_ae_ values between idiopathic and focal epilepsies. High values of T_ae_ were found in patients who had seizures every day and in whom the attacks were poorly controlled by anticonvulsant therapy. It was also found that the value of T_ae_ during the seizure was not higher than in the interictal period. This indicated that the low water permeability of RBCs in epilepsy is a permanent alteration (not a transient one). The abnormal water permeability was found in both untreated and treated patients, i. e. was not related to the anticonvulsant therapy. An alteration (decrease) of the permeability to water of RBCs in children with epilepsy was the most likely explanation for the findings. Gh. Benga, Vasile Morariu, Ileana Benga and Cornelia Morariu realized immediately the important significance of findings, as they had already studied extensively the publications regarding the NMR (Vasile Morariu) and epilepsy (Gheorghe and Ileana Benga). In October 1976 a manuscript was sent to Nature, in December 1976 was accepted to be published without change and in February 1977 it appeared (Benga and Morariu, 1977). The authors concluded that „decreased permeability in erythrocytes of epileptics may reflect a membrane defect in all tissues and may be an expression of the individual predisposition in epilepsy; it might be of particular importance in the nervous system. Further studies on erythrocyte membranes in epilepsy may give clues to the understanding of the membrane defect in molecular terms (Benga and Morariu, 1977; Morariu et al., 1981).” Subsequent studies of Morariu and Benga, (1984) on the effects of temperature allowed the calculation of the activation energy (E_a,d_) of the RBC membrane diffusional permeability (P_d_) to water and showed that the water diffusion time (T_e_), is related with P_d_ by the expression involving the cell water volume (V) and the cell surface area (A):

$${\text{P}}_{\text{d}}=\text{ V/A x }1{\text{/T}}_{\text{e}}$$ (1)

Gh. Benga, working at The “Iuliu Haţieganu” University of Medicine and Pharmacy (abbreviated as U.M.F.) Cluj-Napoca, Romania, started an extensive program of research aimed to identify the pathway by which the water molecules cross the membrane. Several important aspects had to be studied until the final goal was achieved: NMR measurements of the effects on P_d_ of various inhibitors and of chemical modifications of membrane proteins, measurements on resealed ghosts (prepared by a special procedure: hemolysis to remove hemoglobin and then restoring the membrane integrity, as described by Schwoch and Passow, 1973), labelling of the protein involved in water permeability by a radioactive inhibitor ^203^Hg-PCMBS, - PCMBS being an abbreviation of *p-*(Chloromercuri) benzenesulfonate, and finally the identification of this protein by SDS-PAGE. After almost a decade of hard work, the water channel protein (WCP) in the human RBC membrane was identified by Gh. Benga’s group. The discovery was reported in two landmark publications (Benga et al., 1986a,b). The discovery of the first WCP was really achieved in 1985 when the first landmark paper was sent for publication to the prestigious American journal *Biochemistry*, which accepted the publication without change (Benga et al., 1986a). In this paper it was stated: „previous labelling experiments with sulfhydryl-reactive reagents did not correlate binding with inhibition of water transport. The binding pattern of PCMBS that was observed in correlation with the inhibition of water diffusion suggests that either or both band 3 and 4.5 proteins could be associated with water channels. Polypeptides migrating in these regions have already been identified in other transport functions, notably anion exchange and the transport of glucose and nucleosides.To date, however, there is no evidence that a specific inhibitor of one of these processes will inhibit water transport. It remains possible that a minor membrane protein that binds PCMBS is involved in water transport.” Finally, it was also indicated how the final confirmation could be achieved. “We believe the best way to clarify the role of bands 3 and 4.5 in water transport will ultimately be through studies on the reconstitution of purified proteins in liposomes.” The second landmark paper was published in 1986 in a well known European journal (Benga et al., 1986b). The title of this paper clearly indicated that Benga’s group has identified the proteins present in the RBC membrane implicated in water transport.

Gh, Benga presented the novelty of the discovery of his group in reviews published before 1990 (Benga, 1989a; Benga, 1989b; Benga, 1989c) and in many publications in the following years (Benga and Borza, 1995; Benga, 2003; Benga, 2009, Benga, 2012a; Benga, 2012b; Benga, 2012c). It should be emphasized that previous “labelling studies” (Brown et al., 1975; Sha’afi and Feinstein, 1977) pointed to band 3 protein (“a major protein”, known to be the anion transporter in the RBC membrane) to also be the water channel. For the first time Gh. Benga considered the possibility that „a minor protein” in the RBC membrane could be a specific water channel.

In 1988 the protein identified by Benga et al. (1986a), Benga et al. (1986b) was by serependipity purified by Peter Agre’s group working at The Johns Hopkins University, School of Medicine, Baltimore, United States (Denker et al., 1988). Agre confessed on several occasions (cited by Alleva et al., 2012): „Our laboratory got into the water channel field by accident”. In 1988 he and his coworkers had no idea of the function of the purified protein, which they called CHIP28, from *CH*annel forming *i*ntegral membrane *p*rotein of *28* kDa (Denker et al., 1988). In addition to the 28 kDa component, the protein had a 35–60 kDa glycosylated component, i.e., the one previously detected by Benga et al. (1986a), Benga et al. (1986b) as the binding site of PCMBS under conditions for the inhibition of water transport across the RBC membrane. In their paper Agre and coworkers (Denker et al., 1988) have cited one of Benga’s group articles (Benga et al., 1983a): „the characteristics of CHIP28 are consistent with other known features of water channels, e.g., CHIP28 proteins in intact RBCs are impervious to proteolytic digestion (Denker et al., 1988; Smith and Agre, 1991) as are water channels (Benga et al., 1983a).” However, they have not cited the two landmark papers previously published by Gh. Benga’s group Benga et al. (1986a), Benga et al. (1986b).

Following the advice of Prof. John C. Parker (Agre’s Clinical Mentor at The Univ. of North Carolina at Chapel Hill) that CHIP28 could be a water channel, Agre’s group performed an experiment which proved that oocytes from *Xenopus laevis* microinjected with in vitro-transcribed CHIP28 RNA exhibited increased osmotic water permeability. This was inhibited by mercuric chloride, therefore, it was suggested that CHIP28 is a functional unit of membrane water channels (Preston et al., 1992). However, they recognized that „the possibility exists that CHIP28 may function as a water channel regulator, rather than the water channel itself.” The final proof that CHIP28 is the water channel itself rather than a water channel regulator was demonstrated by reconstitution in liposomes and direct measurements of osmotic water permeability by the collaboration of Mark Zeidel’s group (from Harvard Medical School) with Peter Agre’s group (Zeidel et al., 1992). This was already suggested by Gh. Benga’s group in the first landmark paper (Benga et al. (1986a).

The protein identified in Cluj-Napoca was the first water channel discovered. Other WCPs were discovered in 1993: in a plant (Maurel et al., 1993) and in the kidney (Fushimi et al., 1993). The name of aquaporins was proposed for the WCPs (Agre et al., 1993) and CHIP28 was named aquaporin 1 (AQP1). In a few years it became obvious that a large family of WCPs exists, with three subfamilies: aquaporins (AQPs), aquaglyceroporins, and S-aquaporins. Moreover, it was discovered that actually the WCP family (with all three subfamilies) belongs to a superfamily of Membrane Intrinsic Proteins (MIPs). MIP is an acronym first used for MIP 26 (Major Intrinsic Protein of 26 kDa) of lens fiber cells in the eye (Gorin et al., 1984). Later, the presence and roles of such proteins in all kinds of species on Terra (from prokaryotes to plants, animals and humans) have been revealed. Lots of papers, special issues of prestigious journals, multi-authored books, were dedicated to the newly discovered proteins (Heymann and Engel, 1999; Benga, 2005; Zardoya, 2005; Gonen and Walz, 2006; Kuchel, 2006; Benga, 2009; Benga, 2012a; Yang, 2017).

In 2003, Peter Agre was awarded the Nobel Prize in Chemistry, which he shared with Roderick MacKinnon for their „discoveries concerning structure and function of channels in cell membranes”.

Agre introduced his Nobel Lecture with these words: „I wish to discuss the background in order to give credit to the individuals who were in this field long before we joined the field. The current view is that the lipid bilayer has a finite permeability for water, but, in addition, a set of proteins exists that we now refer to as “aquaporins”. Their existence was suggested by a group of pioneers in the water transport field who preceeded us by decades–people including Arthur K. Solomon in Boston, Alan Finkelstein in New York, Robert Macey in Berkeley, Gheorghe Benga in Romania, Guillermo Whittembury in Venezuela, Mario Parisi in Argentina–who by biophysical methods predicted that water channels must exist in certain cell types with high water permeability as renal tubules, salivary glands, and red cells (Agre, 2004).” Some comments regarding the 2003 Nobel Prize in Chemistry appeared in 2003 and afterwards (Balaban et al., 2006; Cucuianu, 2006; Haulică, 2006).

George Emil Palade (1974 Nobel Laureate in Physiology of Medicine) sent on December 2003 a message by fax supporting the recognition of the priority of Gh. Benga:

“Dear Doctor Benga,

I did not expect The Nobel Committee for Chemistry to select water channels as area to give prominence this year and I did not realize either how close is your work to that of Peter Agre.

The idea of a petition has the merit of attracting the attention to the scientific com-munity in the regrettable mistake of your omission from the group of laureates this year.

In any case I signed the petition received from you, I wish you enough courage and strength to carry through this battle and I remain sincerely,

George E. Palade”

Wed, 19 November 2003 To: Gheorghe Benga <gbenga@clujnapoca.ro> From Naoyuki Taniguchi <proftani@biochem.med.osaka-u.ac.jp> Subject: I regret very much.

Dear Professor Benga: I was really surprized to know that you are not awarded even though you are the first scientist who discovered aquaporin 1. It is my also great regret to hear that one of the Nobel Laureates did not cite your work which is really unfair. I do not know what kind of politics existed in these processes [...] Sincerely yours, Naoyuki Taniguchi M.D. Ph.D., Professor and Chairman, Dept. of Biochemistry, Osaka Univ. Medical School, Osaka University Graduate School of Medicine, Room B-1, 2-2 Yamadaoka Suita Osaka 565–0871 Japan.

“In the late 1980s, Peter Agre, while working on the rhesus blood group antigens at Johns Hopkins University serependipitously discovered a new membrane protein that he called CHIP28 (*ch*annel *i*ntegral *p*rotein of molecular weight *28* kD). At the time he had no idea that its function was … Previously and independently, Gheorghe Benga and his group in Romania had shown that the water transport inhibitor *p*-chloromercuribenzene sulfonate selectively bound to a protein in red blood cell membranes … Subsequent studies showed that this was a glycosylated form of CHIP28 (Vandenberg and Kuchel, 2003).”

“The detection of water-specific membrane channels in red blood cells belong to the fundamental discoveries in biology of the 20th century … In 1986 and 1988, the independent groups of Gheorghe Benga and Peter Agre, respectively, discovered the water channel proteins which later were called aquaporins (Wolburg et al., 2011).”

“The 2003 Nobel prize in chemistry was awarded for the discovery of “porins”–protein channels that transport molecules through cell membranes. It went to the Americans Peter Agre for aquaporins, or water channels, and Roderick MacKinnon for potassium channels. But aquaporins were first described in 1986 by Gheorghe Benga, in what was then communist Romania. There is no doubt that Agre told us much more about aquaporins than Benga did, but I can’t believe Benga would have been excluded from the award had he been working in a Western nation (Cox, 2014).”

Consequently, looking in retrospect, asking the crucial question, when was the first water channel protein, aquaporin 1, discovered, a fair and clear cut answer would be: the first water channel protein, now called aquaporin 1, was identified or „seen” *in situ* in the human RBC membrane by Benga and coworkers in 1986 (Benga et al. (1986a), Benga et al. (1986b)). It was again „seen” when it was by chance purified by Agre and coworkers in 1988 (Denker et al., 1988), and was again identified when its main feature, the water transport property was found by Agre, Zeidel and coworkers in 1992 (Preston et al., 1992; Zeidel et al., 1992). The discovery of AQP1 laid the ground for the identification of other water channel family members by homology cloning and other means, which has led to the understanding that aquaporins play essential roles in water transport in tissues. Today almost 400,000 articles are indexed under the tag water channel proteins in PubMed (http://www.ncbi.nlm.nih.-gov/pubmed).

## Comparative Light and Scanning Electron Microscopic Aspects of RBCs From Humans and Various Animal Species and NMR Studies of RBC Water Permeability

In September 1989 at an international event on RBCs organized in what was then The “East Berlin” Gh. Benga had the chance to meet Professor Philip Kuchel (The University of Sydney), who was aware of the papers published in 1977 by Benga and Morariu (mentioned above). The idea of a comparative program of studies of water permeability of RBCs in various animals occurred in the discussion. Gh. Benga mentioned that in Cluj-Napoca such studied have already been started and it would be very interesting to compare the characteristics of water permeability of RBCs from animal species living in Europe with those living only in Australia or introduced from Europe to Australia. A collaborative program of research of Gh. Benga’s group in Romania with Philip Kuchel, Guy Cox and other distinguished Australian scientists whose names are listed in the Dedication (The Australian group) was established after 1990, when the “communist” regime collapsed in Romania. The two groups achieved exchange working visits, performing studies of the RBC water permeability of over 30 species and the program is still active.

Samples of blood were obtained from: „Iuliu Haţieganu” University of Medicine and Pharmacy Cluj-Napoca, Romania; Taronga Zoo, Sydney, NSW; Dubo Zoo, NSW; University of New England, Armidale, NSW; CSIRO McMaster Laboratory, Sydney, NSW; CSIRO Wildlife and Ecology Division, Canberra, ACT; Department of Veterinary Physiology, University of Sydney, NSW.

Species studied were: **Placentals:** man (*Homo sapiens*), mouse (*Mus musculus*), rat (*Rattus norvegicus*), sheep (*Ovis aries*), dog (*Canis familiaris*), dingo (*Canis lupus dingo*), horse (*Equus ferus caballus*), cow (*Bos taurus*), guinea pig (*Cavia porcellus*), rabbit (*Chinchilla*) (*Oryctolagus cuniculus*), alpaca (*Lama pacos*), camel (*Camelus dromaderius*), elephant (*Elephas maximus*). **Marsupials:** bilby **(** *Macrotis lagotis sagitta*), bandicoot (*Isoodon macrourus*), Tasmanian devil (*Sarcophilus harrisii*), koala (*Phascolarctus cinereus*), brushtail possum (*Trichosurus vulpecula*), Godfellow’s tree kangaroo (*Dendrolagus goodfellowi*), Bennett’s wallaby (*Macropus rufogriseus*), parma wallaby (*Macropus parma*), swamp wallaby (*Wallabia bicolor*), tammar wallaby (*Macropus eugenii*), whiptail wallaby (*Macropus paryi*), Eastern grey kangaroo (*Macropus giganteus*), red kangaroo (*Macropus rufus*). **Monotremes:** platypus (*Ornithoryncus anatinus*), echidna (*Tachyglossus aculeatus*). **Birds:** little penguin (*Eudyptula minor*), chicken (*Gallus domesticus*). **Reptiles**: green sea turtle (*Chelonia mydas*), saltwater crocodile (*Crocodyllis porosus*). Human RBCs were used as reference materials.

Blood was collected into heparinised tubes (∽15 IU/ml), refrigerated within 30 min and used within 72 h. The RBCs were isolated by centrifugation, washed three times in medium S (150 mMNaCl, 5.5 mM glucose, 5 mM Hepes [4-(2-hydroxy-ethyl)-1-piperazine ethanesulphonic acid), pH 7.4. Finally, the erythrocytes were suspended in medium S (supplemented with 0.5% bovine serum albumin) at a hematocrit of 30–50%.

The mean cell volumes were calculated from the measurements of hematocrits and mean cell counts, using a Sysmex-CC 130 Microcell counter (Tao Medical Electronics Co. Ltd., Kobe, Japan).

The cell water content was determined by drying samples of RBCs at 105°C to constant weight (∽15 h) and calculating the cell water volume as a fraction of cell volume.

The cell surface areas were calculated from the mean cell diameters when the cells were swollen to spheres in hypotonic NaCl solutions containing 0.5% (w/v) albumin as previously described (Benga et al., 1993a). The measurements were performed using an image analyzer (Tracor Northern TN 8502, Madison, WI, United States).

For scanning electron microscopy (SEM), samples of sedimented washed RBCs were fixed using 1% glutaraldehyde in medium S. After 90 min at 0^°^C the cells were sedimented and washed twice in 150 mM-phosphate buffer, pH 7.2. They were then post-fixed in 1% osmium tetroxide, dehydrated and critical-point dried from CO_2_. After mounting and sputter-coating with gold, samples were examined and photographed in a Hitachi HU-11A (in Romania) and a Jeol JSM-6300 f scanning electron microscope (in Australia). The diameters of RBCs were measured on photographs using a binocular enlarging system with a calibrated eye piece. The measurements were performed only on cells lying completely flat or exactly on edge. Other details of the SEM analyses were previously described (Benga et al., 1992b; Benga et al., 1999; Benga et al., 2000a; Benga et al., 2003; Benga et al., 2010a; Benga et al., 2010b; Benga et al., 2015).

**FIGURE 2:**
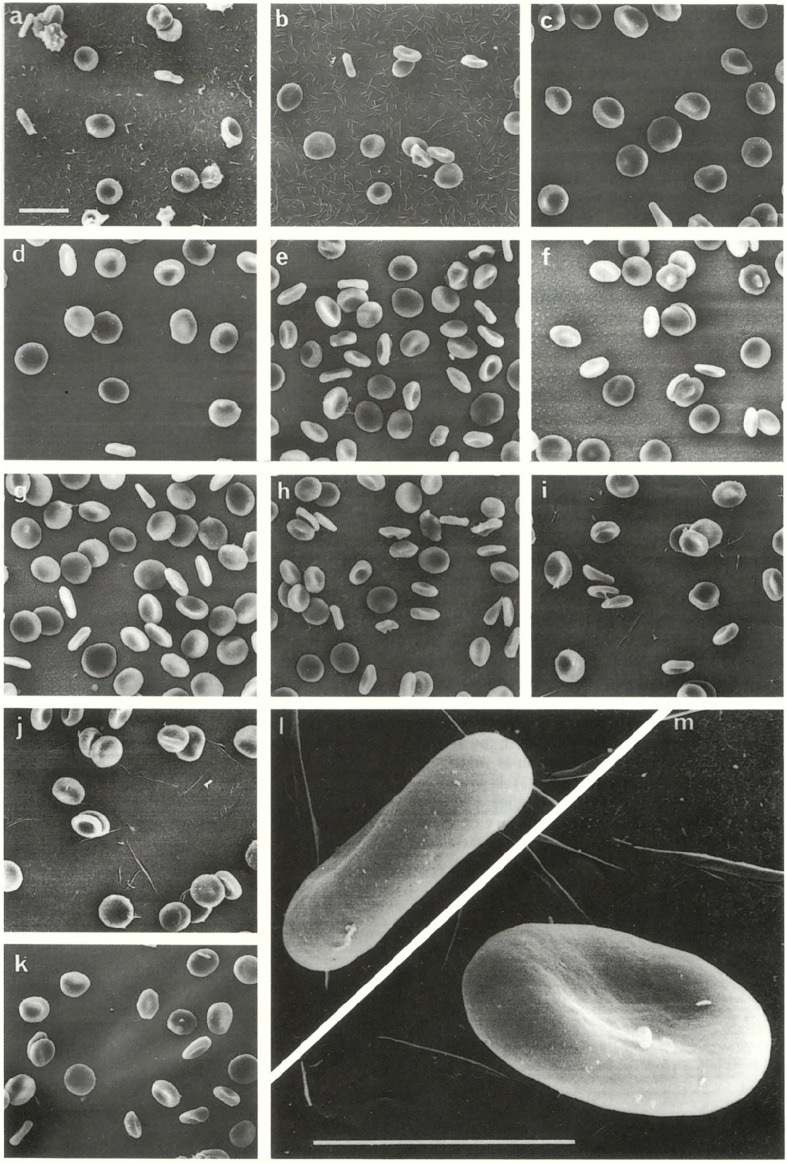
Marsupials. Scanning electron microscopic appearance of red blood cells from **(A)** bandicoot; **(B)** bilby; **(C)** koala; **(D)** red kangaroo; **(E)** Bennett’s wallaby; **(F)** parma wallaby; **(G)** swamp wallaby; **(H)** Tammar wallaby; **(I,J)** whiptail wallaby; and **(K–M)** man. **(A–K)** original magnification ×2,000, scale bar 5 µm; l, m original magnification ×10,000, scale bar 5 µm. The original images were published by (Benga et al., 1992b).

**FIGURE 3:**
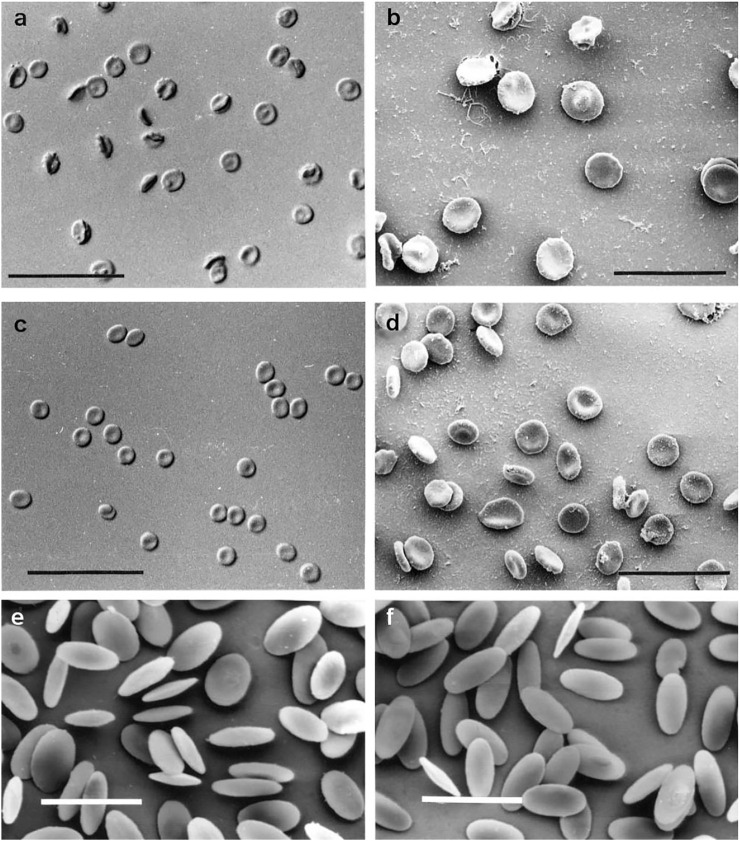
**(A)** DIC optical micrograph of Indian elephant (*Elephas maximus*) RBCs. Scale bar = 50 µm (Nikon Eclipse E800 Plan Apo ×40 0.95NA objective); **(B)** Secondary electron SEM image of Indian elephant RBCs. Scale bar = 22 µm (Jeol JSM-6300f); **(C)** DIC optical micrograph of human (*Homo sapiens*) RBCs. Scale bar = 50 µm (Nikon Eclipse E800 Plan Apo ×40 0.95NA objective.); **(D)** Secondary electron SEM image of human RBCs. Scale bar = 22 µm (Jeol JSM-6300f); **(E)** Secondary electron SEM image of washed RBCs from camel (*Camelus dromedarius*). Scale bar = 10 µm (JEOL JSM-6300f); **(F)** Secondary electron SEM image of washed RBCs from alpaca (*Lama pacos*). Scale bar = 10 µm (JEOL JSM-6300f). The original images were published by Benga et al., 1999; Benga et al., 2000a.

A selection of optical micrographs and SEM images of RBCs from humans and some animal species are presented in Figures 1–3. Some aspects of RBCs from these Figures need to be discussed. A first important aspect is the *presence of nucleus* in the RBCs of reptiles and birds: Green Sea Turtle (*Chelonia mydas*), respectively Little Penguin (*Eudyptula minor*) presented in Figure 1. As mentioned in the Introduction, P. Plósz, (1871) reported the presence of „nuclein” in the hemolyzed nucleated erythrocytes from birds and snakes and this was actually a crucial step in the discovery of DNA.

The second aspect is the correlation between the *size* of RBCs with the whole *body size* (*mass*) of various organisms. In Figure 2 RBCs from nine species of marsupials are presented: bandicoot (*Isoodon macrourus*), bilby (*Macrotis lagotis sagitta*), koala (*Phascolarctus cinereus*), red kangaroo (*Macropus rufus*), Bennett’s wallaby (*Macropus rufogriseus*), parma wallaby (*Macropus parma*), swamp wallaby (*Wallabia bicolor*), Tammar wallaby (*Macropus eugenii*), whiptail wallaby (*Macropus paryi*). The RBCs of these species have a rather similar size, although the whole body size is highly variable (from the small sizes in bilby and bandicoot to the large sizes of wallabies and kangaroos). The relationship between the size of RBCs and the body size (mass) is also easy to be seen in the case of the Indian elephant (*Elephas maximus*) compared with humans (*Homo sapiens*) (Figure 3). The diameter of the elephant RBC is ∽ 9.3 μm, which is ∽1.4 μm larger than that for the human RBC (Benga et al., 2000a). The difference between the whole body size (mass) of these two species is huge. As mentioned in the Introduction such observations led to the so called “Law of constant volume”, formulated in the 19^th^ century (De Robertis, 1970).

The third aspect is the *shape* of RBCs. The human RBCs, and the RBCs of the majority of animal species are biconcave disks. This is true for: 1) the RBCs of monotremes: platypus (*Ornithorhynchus anatinus*) in Figure 1; 2) the RBCs of all marsupials; 3) the RBCs of some placentals: humans (in Figures 2, 3), Indian elephant (*Elephas maximus*) in Figure 3. On the other hand there are placentals which have ellipsoidal RBCs. This is the case of RBCs from camelids: camel (*Camelus dromedarius*) and alpaca (*Lama pacos*) (Figure 3). It is probably related to their ability to swell rapidly when a dehydrated camel rehydrates and thereby avoid haemolysis (Benga et al., 1999).

Samples of sedimented washed RBCs were fixed using 1% glutaraldehyde in medium S. After 90 min at 0 ^o^C the cells were sedimented and washed twice in 150 mM-phosphate buffer, pH 7.2. They were then post-fixed in 1% osmium tetroxide, dehydrated and critical-point dried from CO_2_. After mounting and sputter-coating with gold, samples were examined and photographed in a Hitachi HU-11A (in Romania) and a Jeol JSM-6300 f scanning electron microscope (in Australia). The diameters of RBCs were measured on photographs using a binocular enlarging system with a calibrated eye piece. The measurements were performed only on cells lying completely flat or exactly on edge. The details of the SEM analyses were previously described (Benga et al., 1992b; Benga et al., 1999; Benga et al., 2000a; Benga et al., 2003; Benga et al., 2010a; Benga et al., 2010b; Benga et al., 2015). The values are mean ± standard deviations.

Samples of elephant (*Elephas maximus*) blood were obtained from Taronga Zoo, Sydney, New South Wales; the donor was a female, aged 43 years and weighing 3500 kg. Blood samples were collected into heparin (15 IU/ml), refrigerated within 30 min and used within 72 h. The last sample had a haematocrit of 48%, mean whole-blood haemoglobin concentration of 172 g/l whole blood, a mean corpuscular haemoglobin concentration of 358 g/l RBC and a mean corpuscular volume of 126 fl. The RBCs were isolated by centrifugation, washed three times in medium S (150 mMNaCl, 5.5 mM glucose, 5 mM Hepes [4-(2-hydroxy-ethyl)-1-piperazine ethanesulphonic acid)], pH 7.4. Finally, the erythrocytes were suspended in medium S (supplemented with 0.5% bovine serum albumin) at a hematocrit of 30–50%. For light microscopy, samples of RBCs were fixed for 90 min in 1% (w/v) glutaraldehyde in with medium S, followed by three washes in isotonic phosphate buffer, pH 7.2. The suspended cells were then placed on a clean microscope slide, a cover slip was placed over them and they were examined using a Nikon Eclipse 800 microscope (Nikon Corporation, Tokyo, Japan) using the differential interference contrast (DIC) technique. Image acquisition was performed using a charge-coupled device (CCD) Imaging Sensicam (PCO Computer Optics, GmbH, Kelheim, Germany) at 1280 × 1024 pixels. A stage micrometer was used as a size reference. For scanning electron microscopy (SEM) the samples were prepared as described above and examined and photographed using a Philips XL30 scanning electron microscope. The results are presented in Tables 1, 2. The original results were published by Benga et al., 1992b; Benga et al., 2000a.

**TABLE 1:** Diameters of animal RBC compared with human RBC, measured by electron microscopy.

Species	Number of cells measured	Diameter (µm)	
		Observed	Corrected
Bandicoot	40	4.66 ± 0.15	7.12 ± 0.22
(*Isoodon macrourus)*			
Bilby	70	4.59 ± 0.23	7.01 ± 0.36
(*Macrotis lagotis sagitta)*			
Tasmanian devil	24	4.43 ± 0.19	6.77 ± 0.29
(*Sarcophilus harrisii*)			
Koala	45	5.63 ± 0.20	8.60 ± 0.31
(*Phascolarctus cinereus*)			
Bennet’s wallaby	71	5.27 ± 0.15	8.05 ± 0.23
(*Macropus rufogriseus*)			
Parma wallaby	108	5.23 ± 0.19	8.00 ± 0.29
(*Macropus parma*)			
Swamp wallaby	108	5.61 ± 0.15	8.57 ± 0.23
(*Wallabia bicolor*)			
Whiptail wallaby	37	5.48 ± 0.18	8.38 ± 0.27
(*Macropus paryi*)			
Tammar wallaby	98	5.07 ± 0.12	7.76 ± 0.18
(*Macropus eugenii*)			
Goodfellow’s tree kangaroo	37	4.82 ± 0.14	7.30 ± 0.21
(*Dendrolagus goodfellowi*)			
Red kangaroo	75	5.46 ± 0.11	8.35 ± 0.17
(*Macropus rufus*)			
Man	77	5.24 ± 0.18	8.00 ± 0.22
(*Homo sapiens*)			

**TABLE 2:** Diameter of elephant RBC compared with human RBC.

Species	Technique	Number of cells	Diameter (µm)	Corrected
		Measured	Observed	
Elephant	Light microscopy	26	9.3 ± 0.7	
	SEM	46	7.8 ± 0.6	9.3 ± 0.7
Man	Light microscopy	29	8.0 ± 0.4	
	SEM	31	6.7 ± 0.4	8.0 ± 0.6

**FIGURE 4:**
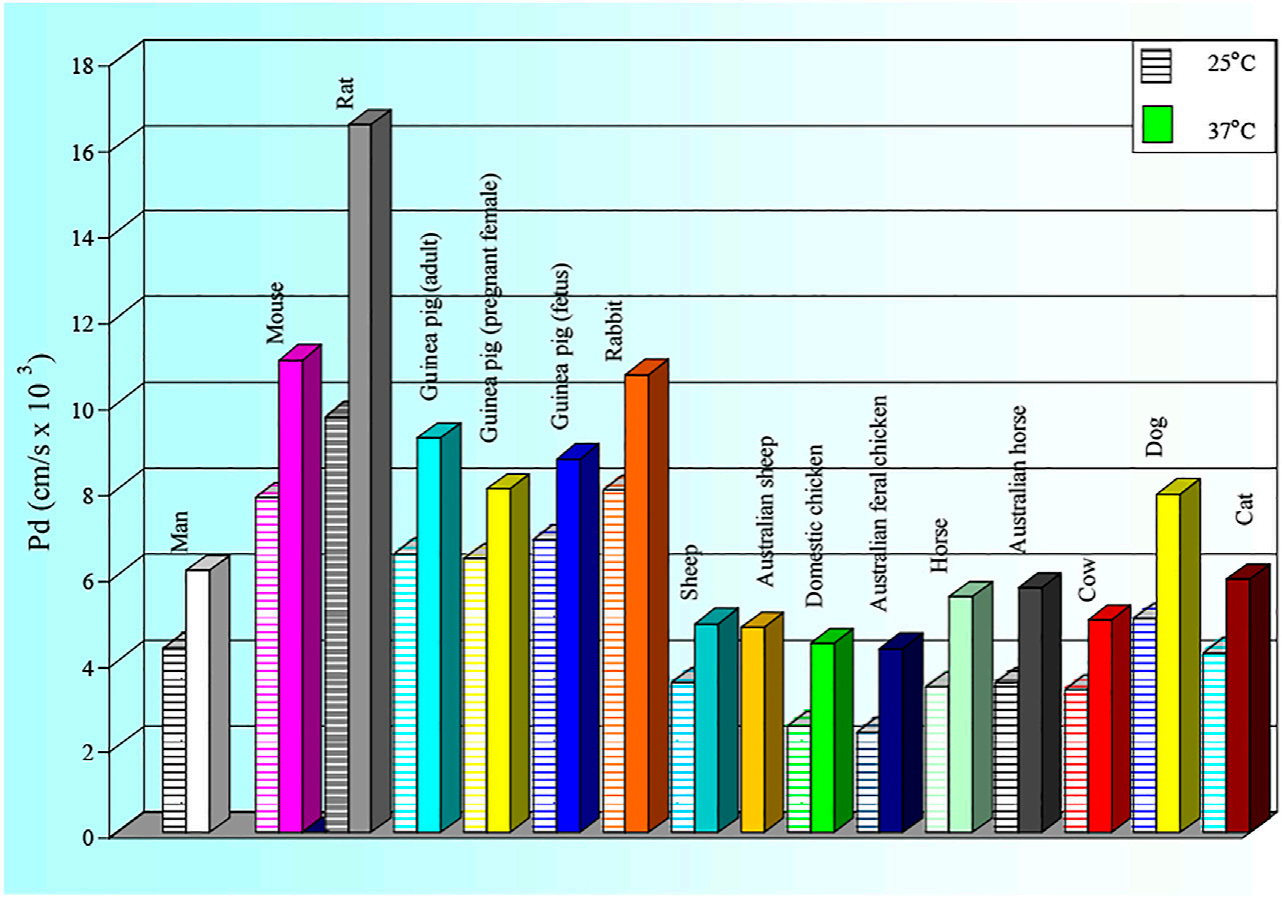
Values of the membrane diffusional permeability (P_d_) to water of RBCs of man and several animal species. The SD values are: Man: 25 ^0^C: 0.50; 37 ^0^C: 0.12; Mouse: 25 ^0^C: 0.30; 37 ^0^C: 0.81; Rat: 25 ^0^C: 0.23; 37 ^0^C: 0.21; Guinea pig (adult).25 ^0^C: 1.31; 37 ^0^C: 2.02; Guinea pig (preganant female): 25 ^0^C: 1.31; 37 ^0^C: 2.02; Guinea pig (fetus): 25 ^0^C: 0.66; 37 ^0^C:1.16; Rabbit: 25^0^C: 0.68; 37 ^0^C:1.77; Sheep: 25 ^0^C: 0.54; 37 ^0^C: 0.86; Australian sheep: 25 ^0^C: 0.75; 37 ^0^C:1.06; Domestic chicken: 25 ^0^C: 0.25; 37 ^0^C: 0.21; Australian feral chicken: 25 ^0^C: 0.37; 37 ^0^C: 0.55; Horse 25 ^0^C: 0.87; 37 ^0^C: 0.75; Australian horse: 25 ^0^C: 1.07; 37 ^0^C: 1.02; Cow: 25 ^0^C: 0.54; 37 ^0^C: 0.86; Dog: 25 ^0^C: 0.20; 37 ^0^C: 0.22; Cat 25 ^0^C: 0.50; 37 ^0^C:0.72.

**FIGURE 5:**
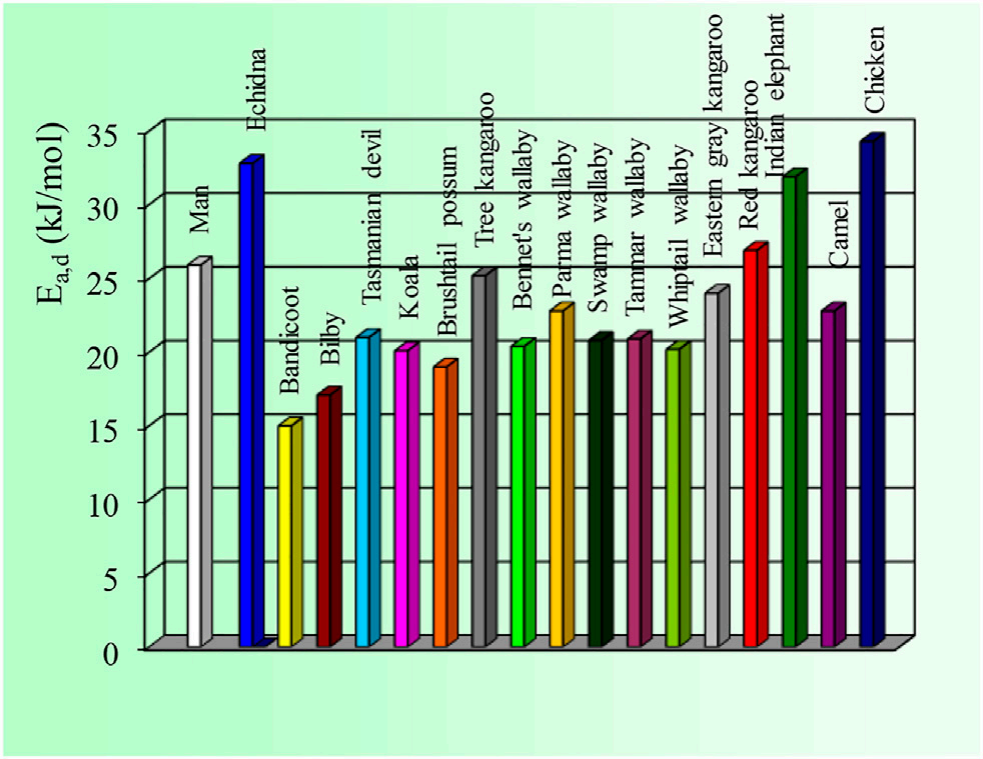
Values of the activation energy of water diffusion through the RBC membrane of man and several animal species. The SD values are: Man: 2.9; Echidna: 6.2; All Marsupials: 1.9–2.0; Elephant: 0.6: Camel: 1,8: Chicken: 7.0. The original results were published previously (Benga et al., 1992b; Benga and Borza, 1995; Benga et al., 1999; Benga et al., 2000a; Benga et al., 2003; Benga et al., 2010a; Benga et al., 2010b; Benga, 2013; Benga et al., 2015).

**FIGURE 6:**
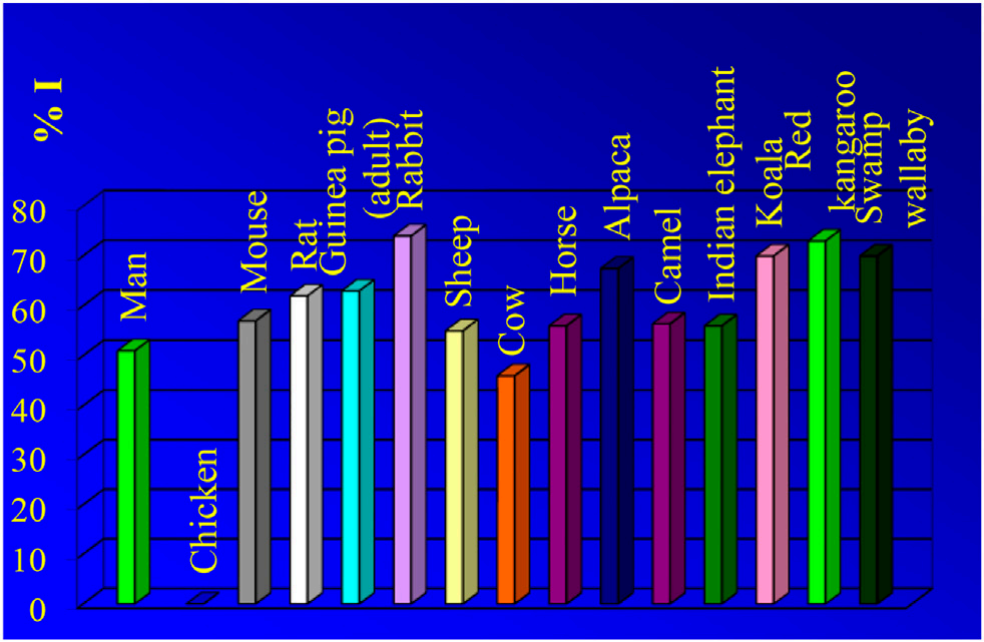
Effects of PCMBS (inhibitor) of water channel proteins on water diffusion through the RBC membrane of man and several animal species. The highest value of inhibition is presented for each species. The original results were published previously (Benga et al., 1992b; Benga and Borza, 1995; Benga et al., 1999; Benga et al., 2000a; Benga et al., 2003; Benga et al., 2010a; Benga et al., 2010b; Benga, 2013; Benga et al., 2015).

The results of our comparative NMR studies of water permeability of RBCs from humans and various animal species were published in many papers (Benga et al., 1992a; Benga et al., 1992b; Benga et al., 1993a; Benga et al., 1993b; Benga et al., 1993c; Benga et al., 1993d; Benga et al., 1993e; Benga et al., 1994a; Benga et al., 1994b; Benga et al., 1995; Benga and Borza, 1995; Benga et al., 1996; Benga et al., 1999; Benga et al., 2000a; Benga et al., 2000b; Benga et al., 2002a; Benga et al., 2002b; Benga et al., 2003; Benga et al., 2009; Benga et al., 2010a; Benga et al., 2015). We express here (in Figures 4–6) only a brief overview of our studies, previously published (Benga et al., 1992b; Benga and Borza, 1995; Benga et al., 1999; Benga et al., 2000a; Benga et al., 2003; Benga et al., 2010a; Benga et al., 2010b; Benga, 2013; Benga et al., 2015).

As shown in Figure 4 and Figure 5 the RBC water permeability (P_d_ and E_a,d_) are species characteristics, as there are no changes correlated with the marked alteration in the habitat of the species introduced to Australia (rat, rabbit, sheep, chicken) compared with their European counterpart. Human RBCs have P_d_ values of ∼4 × 10^–3^ cm s^−1^ at 25 ^0^C and ∼7 × 10^–3^ cm s^−1^ at 37 ^0^C with a value of E_a,d_∼25 kJ mol^−1^. The chicken and echidna RBCs have the lowest P_d_ values (∼2 × 10^–3^ cm s^−1^) and the highest values of E_a,d_ (over 30 kJ mol^−1^). This indicates that no functional AQPs are present in chicken and echidna RBCs. Large and less-active animals (cow, sheep, horse and elephant) have lower values of P_d_. In contrast, small and active animals (mouse, rat, guinea pig, rabbit, small marsupials) have P_d_ values significantly higher with lower E_a,d_ values (from 15 to 22 kJ mol^−1^).

The original results were published previously (Benga et al., 1992b; Benga and Borza, 1995; Benga et al., 1999; Benga et al., 2000a; Benga et al., 2003; Benga et al., 2010a; Benga et al., 2010b; Benga, 2013; Benga et al., 2015).

We measured the effects of various inhibitors on water diffusion through the RBC membrane of man and several animal species. As previously described, the water channels are blocked by PCMBS: *p-*(Chloromercuri) benzenesulfonate. As shown in Figure 3 PCMBS has no effects in case of chicken RBCs. This indicates that no functional AQPs are present in chicken RBCs.

Kuchel and Benga, (2003), Kuchel and Benga, (2005) provided two new explanations for the physiological „raison d’etre” of AQPs in RBC. The first is the „oscillating sieve explanation”: the high water permeability of RBC membrane favours the energy driven membrane undulations (or oscillations) of the RBC membrane, a phenomenon also called „flickering” (Morariu et al., 1966; Brochard and Lennon, 1975); these movements consume a minimum of energy in simply displacing water. Such membrane undulations perform a valuable role in movement of cells through capillaries. The second is the “water displacement explanation”: when ions, such as Cl^**−**^ and HCO_3_, and solutes, such as glucose, are entering into the cells, the water molecules are displaced and exit rapidly the cell, thus obviating a change in cell volume. The molecular volume of these ions and molecules are significantly higher than that of water. Kuchel and Benga, (2003), Kuchel and Benga, (2005) that AQPs in RBCs ensure the rate of exchange of water across the membrane required in various animals in relation to their physical activity, metabolic rate and the mean rate of circulation of their blood. Whether there is a correlation between the macroscopic whole-body activity and the cellular-membrane fluctuations, and hence the requirement (according to the above hypothesis) for differences in water exchange rate, is as yet unknown, and begs new investigations.

## Conclusion

Many discoveries in cell biology have been based on RBCs. Advances in molecular and structural biology in the past 40 years, have enabled the discovery with these cells, most notably, of the water channel protein, called today aquaporin1 (AQP1). It appears that AQPs in RBCs ensure the rate of exchange of water across the membrane required in various animals in relation to their physical activity, metabolic rate and the mean rate of circulation of their blood.

## Dedications

We dedicate this paper to the people from Australia (Bogdan E. Chapman, Clifford H. Gallagher), Romania (Adriana Hodȃrnău, Victoria Borza, Vasile V. Morariu, Dorin Poruţiu, Petre T. Frangopol) and United Kingdom (John Wrigglesworth, William Ferdinand), who contributed essentially to the work on RBC water permeability over the decades and have now passed away.
